# Top 50 Cited Articles on the Treatment of Major Depressive Disorder

**DOI:** 10.7759/cureus.107337

**Published:** 2026-04-19

**Authors:** Ahmed A Albesher, Nayef A Muflehi, Yazan A Hakami, Jana A Barnawi, Randah E Softah, Luluwah H Al Ghalib, Mayar A Bukhari, Reem A Almotiri, Maha S Algethami, Shahad W Mahboob, Relam K Hakami, Ali Alsaad

**Affiliations:** 1 College of Medicine, King Faisal University, Al Hofuf, SAU; 2 College of Medicine, Arabian Gulf University, Manama, BHR; 3 College of Medicine, Royal College of Surgeons in Ireland, Dublin, IRL; 4 College of Medicine, King Saud Bin Abdulaziz University for Health Sciences, Jeddah, SAU; 5 College of Medicine, King Abdulaziz University, Jeddah, SAU; 6 College of Medicine, Majmaah University, Majmaah, SAU; 7 College of Medicine, Batterjee Medical College, Jeddah, SAU; 8 Department of Neuroscience, King Faisal University, Al Hofuf, SAU

**Keywords:** antidepressants, major depressive disorder, mdd, psychotherapy, treatment

## Abstract

Major depressive disorder (MDD) is a common mental health disorder and is one of the leading causes of disability. Numerous treatment modalities have been studied for MDD, from interventions to psychotherapies. However, no exhaustive investigation has yet provided an overview of influential research on MDD treatments. Studies do not cover influential efforts occurring in the form of a comprehensive bibliometric review. This bibliometric study aims to fill this gap by thoroughly examining the characteristics of the 50 most cited articles on MDD treatment and providing an essential understanding of the intellectual structure and historical development over time. To guarantee a comprehensive research collection of trends in the treatment of MDD, we reviewed 50 pertinent papers without limiting the search by publication year, and the data collection was performed in a neutral manner. The search focuses on extensive citations from other publications and categorizes them according to their frequencies. Between 1989 and 2018, the top 50 MDD treatment publications received 96-1,837 citations (median 210, IQR 121-299). The 2000s and 2010s had a high level of publication activity (21 articles each, 42% per decade). Geographically, the United States (24, 48%), Canada (12, 24%), and the United Kingdom (5, 10%) dominated the journals. The majority of study types were randomized controlled trials (RCTs) (32, 64%), followed by systematic reviews/meta-analyses (16, 32%) and prospective cohorts (2, 4%). The publications covered a range of treatment modalities, including pharmacological, psychotherapeutic, neurostimulation, and lifestyle/complementary approaches. Elkin et al.'s NIMH Treatment of Depression Collaborative Research Program report (1,738 citations) and Unützer et al.'s IMPACT collaborative care RCT (1,837 citations) were the most referenced publications. Our results shed light on the conceptual framework of MDD treatment research, as current guidelines are shaped by significant randomized trials and systematic reviews. Precision-based and new treatments are growing the field, while traditional therapies continue to play a major role. Global applicability is limited by the geographic concentration of research, underscoring the need for diverse populations, assessment of new treatments, and incorporation of individual patient data to bolster the body of evidence.

## Introduction and background

Major depressive disorder (MDD) is a severe, debilitating, and highly prevalent mental health condition that ranks among the most disabling disorders worldwide. It is characterized by persistent feelings of sadness, loss of interest in usual activities, and various emotional and physical symptoms that interfere with everyday functioning [1]. Globally, MDD affects a substantial proportion of the population, with a point prevalence of 4.7% and a pooled annual incidence of 3.0%, reflecting its widespread and ongoing impact [2]. It is also one of the leading causes of disability, contributing considerably to the global burden of disease and years lived with disability (YLDs) across all age groups and regions [3]. Furthermore, approximately 16.6% of adults experience depression at some point in their lives, highlighting its long-term prevalence and serious public health implications [4].

In response to this burden, a range of treatment approaches has been developed, including pharmacological options such as antidepressants and mood stabilizers, psychotherapeutic methods such as cognitive behavioral therapy (CBT), and neuromodulation techniques such as electroconvulsive therapy (ECT) [5,6]. However, despite decades of research on MDD’s pathogenesis and treatment, approximately 30% of patients remain treatment-resistant, presenting ongoing challenges in clinical practice [7,8]. This persistent gap highlights the need to better understand the landscape of MDD treatment research.

A substantial amount of research has been published on the treatment of MDD, with the majority primarily focused on synthesizing clinical evidence through systematic reviews (SR) and meta-analyses (MA) [9-11]. These comprehensive studies have provided strong evidence for both pharmacological and psychological interventions, effectively guiding clinical practice by establishing the efficacy and acceptability of various treatment options. In addition to these clinical syntheses, a few bibliometric analyses have been conducted on MDD. However, these have largely been limited to narrower areas of the literature, such as resting-state functional MRI (fMRI) research or single treatment modalities like drug therapy [12,13]. Accordingly, an important gap remains in the literature, as influential MDD treatment research across multiple therapeutic modalities has not yet been examined bibliometrically.

This gap is important because it limits quantitative understanding of the intellectual structure of the field, the evolution of research themes, and the contributions of key authors and institutions. Bibliometric analysis offers a useful way to address this by applying quantitative methods to publication and citation data in order to assess influential publications, citation impact, and broader research trends within a field [13-15]. In addition, it can provide quantitative insights into research patterns, collaboration networks, and scientific impact that are not captured by traditional clinical reviews [13-15]. In MDD treatment research, this approach can help identify landmark studies that have shaped clinical thinking, informed guideline development, and influenced subsequent research directions. Within this context, focusing on the 50 most cited articles provides a pragmatic and manageable overview of influential research while keeping the dataset focused and interpretable.

Therefore, this study aims to address this gap by conducting a bibliometric analysis of the 50 most cited articles on the treatment of MDD. By analyzing their characteristics, study designs, and overall citation influence, we seek to identify influential publication patterns, emerging research trends, and the intellectual development of MDD treatment research.

## Review

Methods

Search Strategy

A thorough literature review utilizing the Web of Science (WoS) Core Collection (Clarivate) database was performed to identify significant papers pertaining to the treatment of MDD. The ranking of the top cited articles was performed within the dataset retrieved from the predefined WoS search query rather than across the entire global literature on depression treatment. The search was executed on July 17, 2025, utilizing the Topic (TS) field tag including titles, abstracts, and keywords. The exact search query was TS= ("Major Depressive Disorder") AND TS=("management"). The search strategy was intentionally designed to capture clinically relevant management-focused literature rather than all publications related to depression treatment, which may include broader pharmacological or mechanistic studies outside the intended clinical scope. No restrictions were applied to the year of publication in order for the entire historical development of the study in this area to be seen. The search was carried out using the WoS Core Collection, which is widely used in bibliometric analyses due to its standardized indexing, comprehensive citation tracking, and consistent bibliographic information, allowing for accurate identification and ranking of highly cited publications. Although PubMed is an extremely useful resource for retrieving scientific publications, it does not give all of the citation data needed to appropriately evaluate highly cited papers, which was critical for the study's objectives. As a result, employing WoS enabled us to conduct a consistent and objective citation-based rating of MDD management articles that was directly related to the goals of this bibliometric analysis. Eligible records were restricted to original research articles, SR, MA, and clinical practice guidelines published in English. The review focused on peer-reviewed journal papers that discussed therapeutic approaches for individuals diagnosed with MDD. The initial search yielded a broad pool of publications. The articles were then ranked based on the total number of times they were cited, as provided by the WoS database. The 50 most cited articles were selected for further analysis, as high citation frequency was seen as a sign of high research influence within the field.

Study Selection

The selection method for the study was conducted independently by four reviewers to reduce selection bias. During the initial screening phase, the titles and abstracts of all retrieved publications were evaluated for relevance according to established inclusion and exclusion criteria. Eligible studies included those focusing on patients with a primary diagnosis of MDD or research investigating MDD in the context of physical comorbidities (e.g., cancer or diabetes), as long as their primary outcome focused specifically on depressive symptom severity, response, or remission. Articles were included if they addressed the treatment of MDD and exhibited substantial academic influence through citations by other scholarly works. Studies were excluded if they were not peer-reviewed or if they consisted of conference proceedings, abstracts, posters, editorials, letters, or commentaries. Discrepancies encountered during the title and abstract screening stages were addressed through discussion. A fifth reviewer was consulted to resolve disagreements when consensus could not be reached among the reviewers. Articles that satisfied the initial screening criteria underwent a comprehensive examination, employing the same methodology, with any remaining discrepancies eventually adjudicated by the senior author.

Data Extraction and Management

Data extraction was performed independently by four reviewers using a standardized form to ensure consistency and reproducibility. The extracted data included the first author's name, year of publication, publication type, total citation count, and the average annual citation rate, which was calculated by dividing the total citation count by the number of years since publication. Citation counts were retrieved from the WoS database on July 17, 2025. Additional extracted data encompassed study characteristics including treatment modality, sample size, mean participant age, study setting, country or region of origin, journal title, journal impact factor, and reported level of evidence. Specifically, the scope of interventions covered a diverse range of therapeutic approaches, categorized into six modalities: conventional pharmacotherapy (e.g., first-line antidepressants), psychotherapy (e.g., CBT, interpersonal psychotherapy, and behavioral activation), and neuromodulation techniques (e.g., ECT and repetitive transcranial magnetic stimulation (rTMS)). Furthermore, this study design included collaborative care, lifestyle interventions (e.g., exercise, yoga, and diet), and adjunctive therapies (e.g., curcuminoids and probiotics), enabling a comprehensive integration of the biological and behavioral landscape of MDD treatment by representing both monotherapy and add-on therapies in the analysis.

Country distribution was determined using a single-country assignment approach based on the corresponding author’s affiliation. Each article was assigned to one country according to the corresponding author’s institutional affiliation. Journal distribution was established by counting the number of included articles published in each journal using the journal titles provided in the WoS database. Before calculating journal frequency distributions, journal titles were manually standardized to address variations in database indexing (e.g., abbreviated titles or alternative naming formats). Records referring to the same journal were harmonized and merged under a single standardized journal name to avoid duplicate counting.

The principal outcomes and key thematic focus of each study were documented. In addition, data extraction focused on standardized clinical outcomes as reported across the included studies to ensure consistency. This included capturing symptom severity through validated tools, primarily the Hamilton Depression Rating Scale (HAM-D), Montgomery-Åsberg Depression Rating Scale (MADRS), and Patient Health Questionnaire-9 (PHQ-9). Additional key outcomes were documented, such as rates of clinical response, remission, functional improvement, and long-term recovery. Discrepancies in data extraction were addressed through discussions among reviewers, with a fifth reviewer consulted as needed to achieve consensus.

The level of evidence for each included study was assessed using the Oxford Centre for Evidence-Based Medicine Levels of Evidence framework [16]. Studies were categorized from Level 1 to Level 5 according to the Oxford Centre for Evidence-Based Medicine Levels of Evidence framework, primarily on the basis of study design. This standardized classification system was applied to ensure consistent evaluation of the strength of evidence across the included publications.

Treatment of Missing Data

When relevant information was missing or inadequately provided in the included papers, efforts were made to reach out to the associated authors for clarification or more data. If authors were unreachable or unresponsive, the absent data were recorded and acknowledged. The potential impact of absent information on the comprehensive analysis and interpretation of data was contemplated and addressed in the discussion of study limitations.

Statistical Analysis

**Figure 1:**
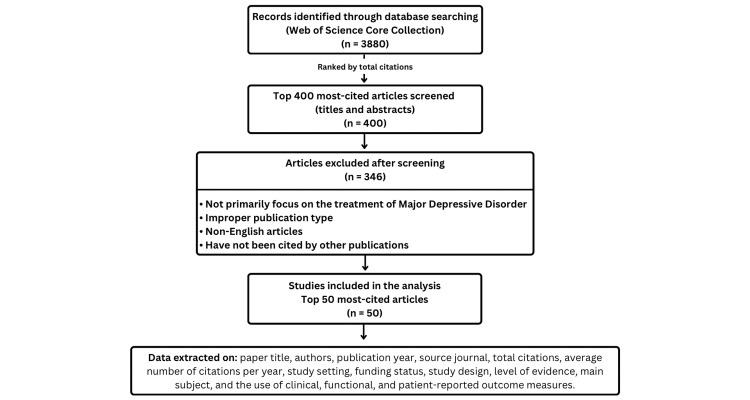
Summary flowchart of our methodology

Descriptive statistical analysis was conducted to encapsulate the attributes of the included research. Continuous variables, including publication year, citation counts, and mean participant age, were summarized utilizing metrics of central tendency and dispersion, such as mean, median, and range. Categorical factors, such as study design, geographic origin, journal type, and treatment method, were summarized with frequencies and percentages. Trends in research influence over time were assessed by examining citation patterns across publication years. Citation counts obtained from the WoS database may include author self-citations and do not distinguish between affirmational and critical citations; therefore, citation metrics were interpreted as indicators of research influence rather than endorsement of study conclusions. A formal quantitative risk-of-bias assessment was not conducted because the primary objective of this study was bibliometric evaluation of citation impact rather than synthesis of clinical outcomes. Nonetheless, a qualitative evaluation of methodological rigor and relevance was conducted during data interpretation to contextualize the findings and identify emerging research trends in the treatment of MDD. An illustration of the screening and selection process is emphasized in the methodological flow chart shown in Figure 1. A total of 3,880 records were identified initially from the WoS database. Then, the records were ranked by total citation counts to focus on the most cited and high-impact literature. Through two stages of screening, stage 1 involved screening the first 200 papers and resulted in 35 articles for full-text assessment, while stage 2 screened the next 200 papers (ranked 201-400), which yielded 19 articles for full-text eligibility.

According to these stages, a total of 346 records were excluded based on specific criteria including articles that do not primarily focus on the treatment of MDD, articles published in non-peer-reviewed sources, published studies in languages other than English, and articles with zero citations. Following the ranking process, four articles (n = 4) were excluded as they did not fall within the top 50 most cited rankings, although they satisfied the clinical eligibility criteria. Ultimately, 50 studies were included in the qualitative synthesis.

Consequently, the final analysis and detailed data extraction was performed on the 50 highest-ranked publications, including bibliometric indicators (title, authors, year, journal, impact factor, and citation metrics), methodological characteristics (design, setting, funding, and level of evidence), and clinical results (subject matter, treatment modalities, and the use of clinical, functional, or patient-reported outcome measures) to ensure a high-quality bibliometric evaluation.

Impact

**Figure 2:**
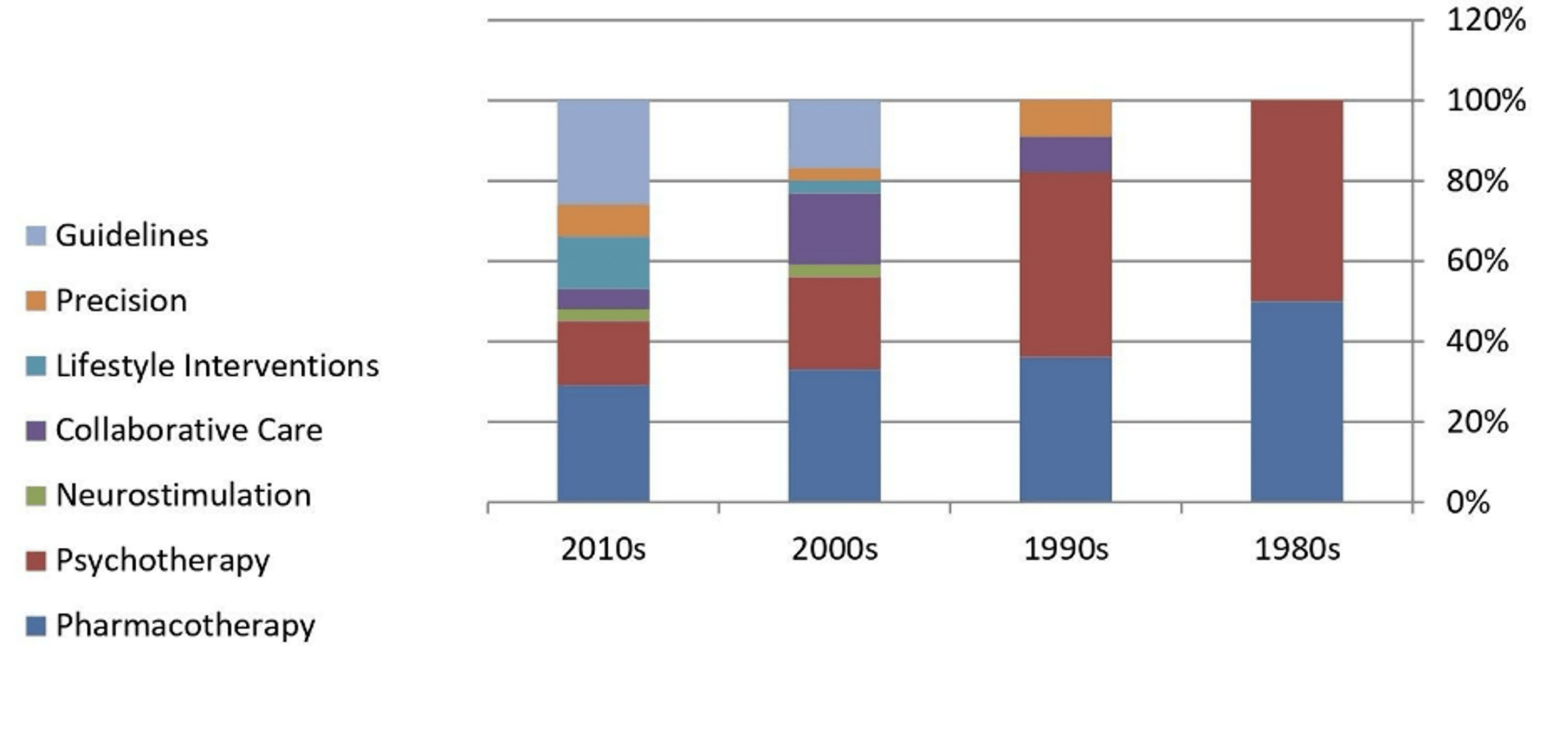
Decade-wise distribution of treatment modalities and research themes among the top 50 cited papers

The top cited clinical and guideline literature on MDD is dominated by landmark randomized controlled trials (RCTs) and large collaborative programs that have shaped contemporary, integrated models of care. Highest citation density is seen for Unützer et al.’s IMPACT trial of collaborative care for late-life depression in primary care (1,837 citations) and the early NIMH Treatment of Depression Collaborative Research Program reported by Elkin and colleagues (1,738 citations), which together established the effectiveness of structured, measurement-based, and team-delivered treatment across settings. Across the list, there is a strong primary care and comorbidity focus (e.g., Katon et al.’s multifaceted primary care intervention, CREATE in coronary artery disease, depression care in diabetes and cancer, and trials in obstetrics/gynecology and VA clinics), reflecting the shift from specialist to integrated, real-world delivery of depression treatment. The repeated appearance of the CANMAT (Canadian Network for Mood and Anxiety Treatments) guidelines (2009 and multiple 2016 sections on pharmacological, psychological, neurostimulation, complementary/alternative, and special populations) underlines their status as global reference standards for evidence-based practice. Several influential trials and MA examine specific modalities and sequencing strategies, including CBT and problem-solving therapy in primary care, behavioral activation, mindfulness-based cognitive therapy, light therapy, rTMS, telephone-administered psychotherapy, and sequential or augmented pharmacotherapy (e.g., aripiprazole augmentation and STAR*D measurement-based care). Special populations receive prominent attention, including older adults, adolescents, and youth/women/elderly subgroups, as well as patients with cancer, cardiovascular disease, diabetes, and irritable bowel syndrome. More recent highly cited works broaden the therapeutic landscape to lifestyle and complementary interventions-dietary improvement (SMILES), exercise, yoga/meditation, meditative movements, curcuminoids, probiotics, and pharmacogenomic-guided prescribing-reflecting a growing precision and personalized-medicine orientation. The top 50 most cited articles on MDD treatment are shown in Table 1. Overall, the citation profile (see Figure 2) demonstrates a measurable evolution from proving individual drug efficacy toward a more nuanced focus on optimized combinations, delivery, and clinical personalization.

**Table 1:** Top 50 by citations (MDD treatment) MDD: major depressive disorder

Rank	Title	Authors	Year of publication	Complete number of citations
1	Collaborative care management of late-life depression in the primary care setting: a randomized controlled trial [17]	Unützer et al.	2002	1,837
2	National Institute of Mental Health Treatment of Depression Collaborative Research Program. General effectiveness of treatments [18]	Elkin et al.	1989	1,738
3	Canadian Network for Mood and Anxiety Treatments (CANMAT) 2016 clinical guidelines for the management of adults with major depressive disorder: section 3. Pharmacological treatments [19]	Kennedy et al.	2016	794
4	A multifaceted intervention to improve treatment of depression in primary care [20]	Katon et al.	1996	634
5	A randomised controlled trial of dietary improvement for adults with major depression (the 'SMILES' trial) [21]	Jacka et al.	2017	595
6	Effects of citalopram and interpersonal psychotherapy on depression in patients with coronary artery disease: the Canadian Cardiac Randomized Evaluation of Antidepressant and Psychotherapy Efficacy (CREATE) trial [22]	Lespérance et al.	2007	467
7	Patient predictors of response to psychotherapy and pharmacotherapy: findings in the NIMH Treatment of Depression Collaborative Research Program [23]	Sotsky et al.	1991	423
8	Course of depressive symptoms over follow-up. Findings from the National Institute of Mental Health Treatment of Depression Collaborative Research Program [24]	Shea et al.	1992	415
9	Canadian Network for Mood and Anxiety Treatments (CANMAT) 2016 clinical guidelines for the management of adults with major depressive disorder: section 4. Neurostimulation treatments [25]	Milev et al.	2016	379
10	Prevention of recurrent depression with cognitive behavioral therapy: preliminary findings [26]	Fava et al.	1998	354
11	Canadian Network for Mood and Anxiety Treatments (CANMAT) clinical guidelines for the management of major depressive disorder in adults. III. Pharmacotherapy [27]	Lam et al.	2009	331
12	Behavioral activation treatments for depression in adults: a meta-analysis and review [28]	Mazzucchelli et al.	2009	302
13	Randomised controlled trial of problem solving treatment, antidepressant medication, and combined treatment for major depression in primary care [29]	Mynors-Wallis et al.	2000	299
14	Antidepressant monotherapy vs sequential pharmacotherapy and mindfulness-based cognitive therapy, or placebo, for relapse prophylaxis in recurrent depression [30]	Segal et al.	2010	288
15	Early improvement in the first 2 weeks as a predictor of treatment outcome in patients with major depressive disorder: a meta-analysis including 6562 patients [31]	Szegedi et al.	2009	284
16	Comparative benefits and harms of second-generation antidepressants for treating major depressive disorder: an updated meta-analysis [32]	Gartlehner et al.	2011	262
17	Initial severity and differential treatment outcome in the National Institute of Mental Health Treatment of Depression Collaborative Research Program [33]	Elkin et al.	1995	258
18	Clinical results for patients with major depressive disorder in the Texas Medication Algorithm Project [34]	Trivedi et al.	2004	249
19	Effect of interventions for major depressive disorder and significant depressive symptoms in patients with diabetes mellitus: a systematic review and meta-analysis [35]	van der Feltz-Cornelis et al.	2010	241
20	Management of depression for people with cancer 4 (SMaRT oncology 1): a randomised trial [36]	Strong et al.	2008	239
21	Canadian Network for Mood and Anxiety Treatments (CANMAT) 2016 clinical guidelines for the management of adults with major depressive disorder: section 2. Psychological treatments [37]	Parikh et al.	2016	236
22	Canadian Network for Mood and Anxiety Treatments (CANMAT) 2016 clinical guidelines for the management of adults with major depressive disorder: section 5. Complementary and alternative medicine treatments [38]	Ravindran et al.	2016	233
23	Improving primary care for depression in late life: the design of a multicenter randomized trial [39]	Unützer et al.	2001	229
24	Aripiprazole augmentation in major depressive disorder: a double-blind, placebo-controlled study in patients with inadequate response to antidepressants [40]	Berman et al.	2009	222
25	Telephone-administered psychotherapy for depression [41]	Mohr et al.	2005	216
26	Randomized controlled trial of collaborative care management of depression among low-income patients with cancer [42]	Ell et al.	2008	203
27	Canadian Network for Mood and Anxiety Treatments (CANMAT) 2016 clinical guidelines for the management of adults with major depressive disorder: section 6. Special populations: youth, women, and the elderly [43]	MacQueen et al.	2016	199
28	Efficacy of bright light treatment, fluoxetine, and the combination in patients with nonseasonal major depressive disorder: a randomized clinical trial [44]	Lam et al.	2016	188
29	Repetitive transcranial magnetic stimulation for treatment-resistant depression: a systematic review and metaanalysis [45]	Lam et al.	2008	188
30	Effects of psychotherapy and other behavioral interventions on clinically depressed older adults: a meta-analysis [46]	Pinquart et al.	2007	176
31	Moderate exercise improves depression parameters in treatment-resistant patients with major depressive disorder [47]	Mota-Pereira et al.	2011	163
32	Effectiveness of collaborative care depression treatment in Veterans' Affairs primary care [48]	Hedrick et al.	2003	161
33	Six-year outcome for cognitive behavioral treatment of residual symptoms in major depression [49]	Fava et al.	1998	160
34	Utility of integrated pharmacogenomic testing to support the treatment of major depressive disorder in a psychiatric outpatient setting [50]	Hall-Flavin et al.	2013	159
35	Escitalopram in the treatment of adolescent depression: a randomized placebo-controlled multisite trial [51]	Emslie et al.	2009	146
36	Investigation of the efficacy of adjunctive therapy with bioavailability-boosted curcuminoids in major depressive disorder [52]	Panahi et al.	2015	146
37	Canadian Network for Mood and Anxiety Treatments (CANMAT) clinical guidelines for the management of major depressive disorder in adults. II. Psychotherapy alone or in combination with antidepressant medication [53]	Parikh et al.	2009	126
38	Using second-generation antidepressants to treat depressive disorders: a clinical practice guideline from the American College of Physicians [54]	Qaseem et al.	2008	121
39	Maximizing the adequacy of medication treatment in controlled trials and clinical practice: STAR(*)D measurement-based care [55]	Trivedi et al.	2007	121
40	Cognitive behavioral therapy for depression in older people: a meta-analysis and meta-regression of randomized controlled trials [56]	Gould et al.	2012	119
41	Canadian Network for Mood and Anxiety Treatments (CANMAT) clinical guidelines for the management of major depressive disorder in adults. IV. Neurostimulation therapies [57]	Kennedy et al.	2009	118
42	Yoga- and meditation-based lifestyle intervention increases neuroplasticity and reduces severity of major depressive disorder: a randomized controlled trial [58]	Tolahunase et al.	2018	108
43	The sequential integration of pharmacotherapy and psychotherapy in the treatment of major depressive disorder: a meta-analysis of the sequential model and a critical review of the literature [59]	Guidi et al.	2016	105
44	The impact of a pharmacist intervention on 6-month outcomes in depressed primary care patients [60]	Adler et al.	2004	105
45	Escitalopram in the treatment of depressed elderly patients [61]	Kasper et al.	2005	103
46	Effects of meditative movements on major depressive disorder: a systematic review and meta-analysis of randomized controlled trials [62]	Zou et al.	2018	101
47	Acute and one-year outcome of a randomised controlled trial of brief cognitive therapy for major depressive disorder in primary care [63]	Scott et al.	1997	98
48	Improving care for depression in obstetrics and gynecology: a randomized controlled trial [64]	Melville et al.	2014	98
49	An intensive treatment program of interpersonal psychotherapy plus pharmacotherapy for depressed inpatients: acute and long-term results [65]	Schramm et al.	2007	97
50	Bacillus coagulans MTCC 5856 for the management of major depression with irritable bowel syndrome: a randomised, double-blind, placebo controlled, multi-centre, pilot clinical study [66]	Majeed et al.	2018	96

Decade Distribution

**Figure 3:**
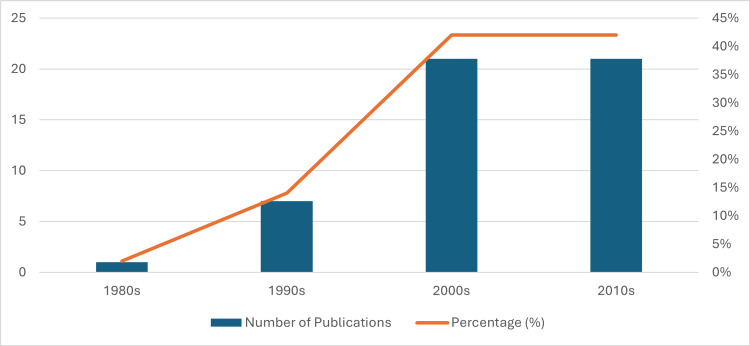
Decade distribution

The percentage distribution of publications across decades shows a clear temporal evolution in influential research on MDD. Only 2% of the top cited articles were published in the 1980s, increasing modestly to 14% in the 1990s, reflecting an early but growing interest in structured treatment trials and collaborative care models. The field then expanded substantially in the 2000s, which, together with the 2010s, accounts for the overwhelming majority of influential work (42% each decade). This sharp rise from 2000 onward suggests both an intensification of research activity and a diversification of study designs, populations, and interventions, coinciding with the development of evidence-based guidelines and integrated care frameworks. Overall, the table indicates that contemporary practice in depression management is largely grounded in evidence generated over the last two decades, highlighting the relatively recent consolidation of the knowledge base guiding current clinical and policy decisions. The distribution of publications across decades is shown in Table 2 and Figure 3.

**Table 2:** Decade distribution table

Decade	Number of publications	Percentage
1980s	1	2%
1990s	7	14%
2000s	21	42%
2010s	21	42%
Total	50	100%

The frequency distribution of journals demonstrates that highly cited research on MDD is concentrated within a small cluster of leading psychiatric outlets, while also spanning a broad multidisciplinary landscape. Archives of General Psychiatry accounts for the highest proportion of the corpus, accounting for 14% of all publications, reflecting its longstanding reputation as a premier venue for landmark clinical trials and etiological research. This is followed by the Canadian Journal of Psychiatry and the American Journal of Psychiatry, contributing 12% and 8%, respectively, underscoring the central role of national psychiatric associations in shaping evidence-based practice. The Journal of Affective Disorders also appears prominently (6%), consistent with its specialization in mood disorder research. A second tier of journals-including JAMA, Annals of Internal Medicine, and General Hospital Psychiatry-each contribute 4%, highlighting an important intersection between psychiatry and general medicine. The remainder of the publications (each 2%) is distributed across a large number of specialized or interdisciplinary journals, spanning geriatrics, oncology, pharmacogenomics, nutrition, psychology, obstetrics, and neuroscience. This wide dispersion of lower-frequency journals suggests that depression research is both clinically broad and methodologically diverse, extending beyond psychiatric settings into wider medical and behavioral health fields. Overall, the table reflects a field anchored by a core set of psychiatric journals while simultaneously engaging multiple adjacent disciplines, reinforcing the multifaceted nature of depression research and treatment. The detailed journal distribution is shown in Table 3.

**Table 3:** Top journals contributing to the 50 most cited MDD treatment articles MDD: major depressive disorder

Name of the journal published in	Count	Percentage
Archives of General Psychiatry	7	14%
Canadian Journal of Psychiatry	6	12%
American Journal of Psychiatry	4	8%
Journal of Affective Disorders	3	6%
Journal of the American Medical Association	2	4%
Annals of Internal Medicine	2	4%
General Hospital Psychiatry	2	4%
Journal of the American Academy of Child and Adolescent Psychiatry	1	2%
Journal of Clinical Psychiatry	1	2%
Phytotherapy Research	1	2%
Journal of General Internal Medicine	1	2%
BMC Medicine	1	2%
BMJ-British Medical Journal	1	2%
Clinical Psychology	1	2%
American Journal of Geriatric Psychiatry	1	2%
CNS Spectrums	1	2%
Journal of Consulting and Clinical Psychology	1	2%
Food & Nutrition Research	1	2%
Journal of Psychiatric Research	1	2%
Lancet	1	2%
Journal of the American Geriatrics Society	1	2%
Medical Care	1	2%
British Journal of Psychiatry	1	2%
Obstetrics and Gynecology	1	2%
Neuropsychopharmacology	1	2%
JAMA Psychiatry	1	2%
Pharmacogenetics and Genomics	1	2%
Aging & Mental Health	1	2%
Restorative Neurology and Neuroscience	1	2%
Journal of Clinical Medicine	1	2%
Journal of Clinical Oncology	1	2%
Grand total	50	100%

Research Setting and Geographic Distribution

**Figure 4:**
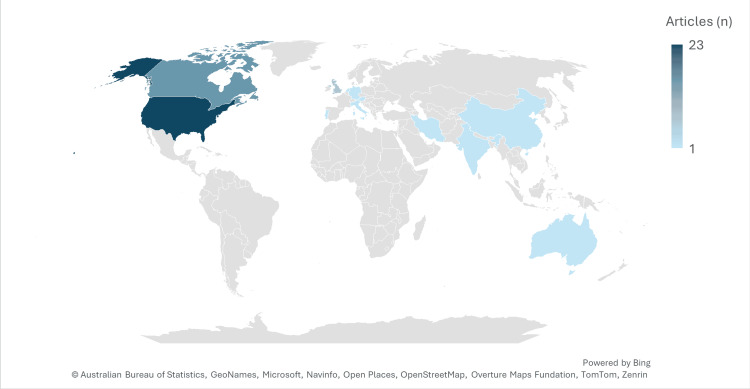
Geographical distribution by country (top 50 set) The color scale represents the number of articles (n), ranging from 1 to 23.

The country distribution shows that influential clinical and guideline research on MDD is heavily concentrated in North America, particularly the United States (48%) and Canada (24%), which together account for nearly three-quarters of all publications. This dominance reflects the strong research infrastructure, funding, and long-standing traditions of large multicenter trials in these settings. The United Kingdom contributes a further 10%, indicating that Anglo-Western, high-income countries are the primary drivers of highly cited depression research. In contrast, contributions from other regions-including Australia, several European countries (Italy, Netherlands, Portugal, Austria, and Germany), and Asian countries (Iran, India, and China)-are each limited to single publications (2% each), suggesting emerging but still modest representation. Overall, the table highlights a pronounced geographic skew toward Western, high-income contexts, which may limit the cultural and health system generalizability of this evidence base and underscores the need for more high-quality research from low- and middle-income countries and diverse sociocultural settings. The geographic distribution of the included studies is shown in Table 4 and Figure 4.

**Table 4:** Geographic distribution by country (top 50 set)

Country	Frequency	Percentage
USA	24	48.0%
Canada	12	24.0%
UK	5	10.0%
Australia	1	2.0%
Italy	1	2.0%
Netherlands	1	2.0%
Portugal	1	2.0%
Iran	1	2.0%
India	1	2.0%
Austria	1	2.0%
China	1	2.0%
Germany	1	2.0%
Total	50	100%

Evidence Hierarchy of the 50 Most Cited MDD Treatment Articles

The distribution of evidence levels according to the Oxford CEBM study-design classification indicates that the body of literature is primarily composed of formal experimental and secondary synthesis designs. RCTs represent the majority of included studies (64%), highlighting a strong reliance on rigorous experimental designs to evaluate interventions for MDD. In addition, SR and MA, which synthesize results across multiple trials, account for 22% of the studies, while a smaller proportion (10%) consists of SR without MA. While these designs are categorized as Level 1 evidence based on the hierarchy of study design, it is important to note that this classification reflects the structural nature of the research rather than a formal quality appraisal or risk-of-bias assessment. Only two studies (4%) were classified as Level 2 evidence, both being prospective cohort designs. Overall, more than 90% of the included publications fall under Level 1 evidence. This predominance of high-level evidence designs underscores the strong empirical foundation supporting current clinical guidelines and treatment recommendations. The hierarchy of evidence among the included studies is shown in Table 5.

**Table 5:** Study designs and levels of evidence of the top 50 most cited MDD treatment articles MDD: major depressive disorder

Study design	Level of the evidence	n (%)
Randomized controlled trials	Level 1	32 (64%)
Systematic review and meta-analyses	Level 1	11 (22%)
Systematic review	Level 1	5 (10%)
Prospective cohort study	Level 2	2 (4%)
Total	-	50 (100%)

Discussion

The bibliometric analysis of the 50 most cited publications on MDD treatment reveals a literature that balances stability with innovation. Our findings indicate a clear dominance of high-level evidence, particularly guideline-driven syntheses and landmark RCTs such as STAR*D and CANMAT, which have established the foundational frameworks for treatment protocols [19,67]. The CANMAT guidelines remain among the most influential frameworks guiding evidence-based treatment of MDD [19,25,27,37,38,43]. The most cited article in the field was “Collaborative care management of late-life depression in the primary care setting: a randomized controlled trial,” which demonstrated the effectiveness of collaborative care in improving depression outcomes among older adults in primary care [17]. The emphasis on pharmacological interventions, psychotherapies, and adjunctive therapies, including exercise and mindfulness, demonstrates an integrated biological and behavioral approach, while emerging research trends in precision psychiatry and novel modalities such as ketamine and rTMS highlight an evolving landscape [68,69].

The top 10 most cited studies account for more than half of all citations, emphasizing the outsized influence of seminal works on clinical guidelines. The 2000s and 2010s represented a pivotal period in the MDD literature, together accounting for 84% of the most cited publications, reflecting a sharp expansion and consolidation of influential research in depression treatment compared with the limited output of earlier decades. North American countries dominate contributions, reflecting the concentration of trials and guideline development in high-resource settings. Nearly all studies (96%) represent Level 1 evidence, reflecting a literature dominated by rigorous RCTs (64%), alongside a substantial contribution from SR and MA (32%). This evidence structure highlights how controlled pharmacological and comparative trials-rather than secondary syntheses alone-drive citation impact and the establishment of clinical standards.

When compared with bibliometric analyses focusing on evidence synthesis rather than primary trials, our findings demonstrate both convergence and complementarity. A recent bibliometric study examining SR and MA on MDD reported a steady increase in publication output from 1983 to 2022, with the United States and the United Kingdom identified as the leading contributors to this body of literature [70]. These geographic patterns closely mirror those observed in our analysis of highly cited original research, reinforcing the central role of high-income countries in shaping the global MDD evidence base [70]. In addition, the thematic clusters identified in the SR/MA literature-particularly those related to clinical interventions, treatment strategies, and brain stimulation-are consistent with the prominent citation clusters observed in our study, suggesting alignment between influential primary trials and subsequent evidence synthesis [70].

However, while bibliometric analyses of SR and MA highlight the rapid expansion and breadth of evidence synthesis in MDD research, our findings underscore the disproportionate influence of a relatively small number of landmark RCTs and guideline-defining studies. This contrast illustrates a hierarchical relationship within the literature, whereby high-impact primary studies establish foundational clinical evidence that is later synthesized and amplified through SR and MA, thereby reinforcing their central role in shaping treatment standards [70].

Despite the historical focus on antidepressants, there is a notable increase in research on non-pharmacological interventions, including neurostimulation and ketamine, signaling a potential shift in research priorities. However, several limitations are apparent. The geographic concentration in high-income, English-speaking countries limits generalizability to low- and middle-income regions. The predominance of North American publications may partially indicate the robust research infrastructure and financial backing in the region, encompassing significant organizations such as the National Institutes of Health (NIH) in the United States and the Canadian Institutes of Health Research (CIHR), which enable extensive clinical trials and impactful publications. The geographic dominance of Western nations must be regarded with caution, as our inclusion criteria limited the research to English-language publications, potentially influencing this distribution. Because the analysis was limited to papers that were indexed in WoS, relevant studies that were indexed in other databases like Scopus, PubMed, or Dimensions would have been overlooked. Because the dataset was derived from a predefined WoS search query using specific topic terms, some highly cited studies addressing depression treatment may not have been captured if they were indexed differently or did not include the selected keywords. Accordingly, the findings should be interpreted within the context of the selected search framework rather than as a representation of the entire global literature on depression treatment. Therefore, the ranking reflects the most cited articles within the retrieved dataset rather than across the entire body of literature on depression treatment. Reliance on a single database may result in coverage bias and favor journals and articles that are primarily indexed in WoS, which may overrepresent Anglo-American research output, because database coverage varies among platforms. The inclusion of various forms of evidence, such as primary studies, SR/MA, and clinical practice guidelines, is one limitation of this bibliometric analysis. Clinical guidelines may somewhat increase the apparent influence of particular studies, journals, or nations because they frequently summarize and reference landmark trials. Therefore, rather than being independent measures of the creation of original evidence in the treatment of MDD, citation counts should be interpreted as indicators of academic influence. Furthermore, only indexed journal articles were included, excluding gray literature [71]. Due to the relatively small dataset (top 50 most cited articles), a keyword co-occurrence network analysis was not performed, as such analyses generally require larger datasets to produce meaningful thematic clustering. One disadvantage of this analysis is the possibility of age bias when ranking articles based on total citation counts, because older publications have had more time to accrue citations. Although average annual citation rates were determined to provide more context on the temporal importance of publications, the primary ranking was based on total citations, which are still the most often used statistic in bibliometric analysis. Alternative metrics like citations per year, field-weighted citation impact, or network-based influence measures might be taken into account in future research.

Future research should focus on conducting contemporary randomized trials across diverse geographic and clinical settings to improve generalizability and on evaluating the effectiveness of novel and adjunctive interventions using rigorous methodological designs. In addition, future bibliometric analyses may further examine geographic contributions by assessing whether the observed distribution persists after excluding guideline publications and by applying fractional counting methods to multinational collaborations, which may provide a more nuanced representation of country-level contributions. Integrating individual patient data from large MA could advance precision psychiatry approaches, and tracking long-term outcomes for psychotherapeutic and adjunctive interventions is necessary to assess relapse prevention and quality of life [72]. Together, these efforts will ensure that the evidence base for MDD treatment continues to evolve in a globally relevant and methodologically robust manner.

## Conclusions

In conclusion, this bibliometric study highlights that the MDD treatment literature is shaped by a small number of highly influential studies, with RCTs and SR forming the core evidence base for clinical guidelines. The field is evolving toward multimodal and precision-based treatments, including ketamine and personalized psychiatry approaches. However, the dominance of research from high-income countries limits global generalizability and underscores the need to diversify study populations and settings. Mapping citation patterns revealed key knowledge gaps and research priorities, emphasizing the importance of expanding geographically representative trials, evaluating emerging therapies, and integrating individual patient data to build a robust, globally applicable evidence base for depression treatment.
